# Adherence to Online Nutritional Monitoring Was Associated with Better Food Habits in People with T1DM during the COVID-19 Pandemic in Brazil

**DOI:** 10.3390/nu15092121

**Published:** 2023-04-28

**Authors:** Daniela Lopes Gomes, Emily Cristina Barbosa da Silva, Aline Leão Reis, Manuela Maria de Lima Carvalhal, Jeane Lorena Dias Kikuchi, Gabriela Correia Uliana, Talita Nogueira Berino

**Affiliations:** 1 Postgraduate Program in Neuroscience and Behavior, Behavior Theory and Research Nucleus, Federal University of Pará, Belém 66075-110, Brazil; a.leaoreis@gmail.com (A.L.R.); nutri.jeanedias@gmail.com (J.L.D.K.); gabriela.ulianaafh@gmail.com (G.C.U.); nutritalitaberino@outlook.com (T.N.B.); 2 Faculty of Nutrition, Federal University of Pará, Belém 66075-110, Brazil; emilycrisilva@gmail.com (E.C.B.d.S.); manuela.carvalhall@gmail.com (M.M.d.L.C.)

**Keywords:** diabetes mellitus, eating habits, social distance, nutritional monitoring

## Abstract

Until this moment, no research has been found that has assessed adherence to online nutritional monitoring by adults with Diabetes Mellitus Type 1 (T1DM) during the pandemic. This article aims to analyze the association between eating habits and adherence to nutritional online care by adults with T1DM during social distancing because of the COVID-19 pandemic in Brazil. A cross-sectional study was carried out in July 2020. An online form was used to collect sociodemographic data, financial status, eating habits, carrying out online nutritional monitoring, and adherence to social distancing. Pearson’s chi-squared test was performed with adjusted residual analysis and binomial logistic regression analysis (*p* < 0.05). Out of the 472 adults, only 8.9% had consulted with a nutritionist. Doing nutritional monitoring online during social distancing was associated with a reduction in the consumption of ultra-processed foods (*p* = 0.021), eating more servings of fruit (*p* = 0.036), and doing carbohydrate counting (CC) more frequently (*p* = 0.000). Doing nutritional monitoring online increased adherence to carbohydrate counting by 2.57 times and increased the consumption of fruits by 0.423 times. Therefore, nutritional monitoring, even if performed remotely, can influence the acquisition and maintenance of healthier eating habits, in addition to assisting adherence to the practice of CC.

## 1. Introduction

Recently, the world has been affected by a disease caused by the SARS-CoV-2 coronavirus called Coronavirus Disease (COVID-19) [1]. In Brazil, there was a declaration of community transmission of COVID-19 in March 2020. COVID-19 is an acute respiratory infection, which can progress to serious and critical conditions, causing cases such as discomfort or respiratory failure, a drop in the saturation of O_2_, multiple organ dysfunction, severe pneumonia, among others [2]. Some chronic diseases are considered risk factors for more severe cases of COVID-19, including diabetes [2,3].

Individuals with diabetes have been greatly affected by the pandemic, given that the measures implemented have included the closure of schools and non-essential bodies and even the cancellation of routine clinical appointments and lockdowns in order to try to contain the spread of COVID-19 [4]. Holman et al. [5] identified that higher levels of glycated hemoglobin (>10%) were associated with a higher risk of death from COVID-19 in people with Diabetes Mellitus Type 1 (T1DM) and 2 (T2DM). Thus, the importance of glycemic control in people with diabetes during this period is reinforced.

The treatment of T1DM should be continuous, with the application of multiple doses of insulin, glycemic self-monitoring, healthy eating, physical activity, and continuous diabetes education for glycemic control to be achieved, as T1DM is caused by the autoimmune destruction of pancreatic beta cells, which generates hyperglycemia if exogenous insulin doses are not applied [6,7,8]. However, it is believed that treatment was impaired during the pandemic due to social isolation, stress, changes in lifestyle, and also a greater difficulty in obtaining supplies and regular face-to-face appointments with health professionals [9,10].

Barone et al. report that even with Brazil taking protective measures such as social distancing, as well as the use of masks and alcohol gel, the population reported numerous fears and challenges; among these are the face-to-face appointments [11,12]. Since the beginning of the pandemic, the obligation of face-to-face care by nutritionists was suspended and online service in Brazil was authorized [13] in order to provide safety to patients and health professionals and allow continuous monitoring or even improvement in the provision of health care [14,15].

However, the challenges in using this system are the lack of experience with online services, technological difficulties, and a lack of access to laboratory reports and contact with the professional (besides virtually) [16]. Zafra-Tanaka et al. [17] described the challenges faced by people living with T1DM to access care during the COVID-19 pandemic in Peru, and discussed the challenges of implementing remote consultations in the health system care, including the difficulty of contacting people with chronic diseases as a result of the lack of updated records of these people, as well as the lack of infrastructure, which includes call centers for scheduling appointments, and equipment and internet services for carrying out appointments.

Despite this, this modality has been valuable for making nutritional assistance possible even with social distancing. Alharthi et al. [18] investigated changes in glycemic control in people with T1D who attended a telemedicine consultation and those who did not attend any consultation during the COVID-19 lockdown period. The authors found that those who attended a consultation had a significant improvement in fasting glucose and glycated hemoglobin, and for those who did not attend any consultation, no alterations were found in these glycemic indices. Thus, there is a clinical efficacy of telemedicine in the treatment of diabetes.

However, access to telemedicine may depend on the patient’s income and place of residence, as these factors may influence internet access and the possibility of paying for consultations in a private service, as health care in public services is rarely carried out in a virtual way, especially in countries such as Brazil, where internet access in rural areas is difficult [19,20].

Olmastroni et al. [21] analyzed, in a systematic review, the main reasons for non- adherence to therapies for chronic diseases during the pandemic: social restrictions and fear of being infected. However, the use of telemedicine is one of the strategies that could improve this adherence, being a viable alternative even after the end of the pandemic restrictions. In Brazil, Binhardi et al. [22] assessed the characteristics of people with diabetes, including self-care practices with diabetes and resilience in the context of the COVID-19 pandemic. The study used an online questionnaire and had a convenience sample of 1633 people with diabetes, including type 1, type 2, and other types of diabetes. The authors found that 31.7% reported telemedicine appointments, having been more frequent in those with T1DM compared to respondents with T2DM. The most frequently consulted professionals were physicians, nutritionists, and psychologists.

The adoption of a healthy eating pattern, prioritizing the ingestion of fresh or minimally processed foods, and avoiding an excessive consumption of high-sugar and ultra-processed foods are some of the most important aspects of glycemic control and the prevention of complications [8,22,23]. In addition, it is important to perform carbohydrate counting to improve glycemic control and allow greater flexibility in eating [8,24] because the amount of carbohydrates ingested is what most increases blood glucose, since 100% of what is consumed in carbohydrates is converted into serum glucose [8]. CC is a dietary method indicated for people with T1DM, as it reduces glycated hemoglobin values compared to a regular diet without CC or alternative dietary methods [25].

In a study by Russo et al. [26], the authors aimed to describe the use of telemedicine by patients with T2DM during the 2020 COVID-19 pandemic in Italy, and observed that telemedicine provided an acceptable quality of diabetes care, comparable to that of patients seen in face-to-face assistance. Scott et al. [27] conducted a global survey on the use of telemedicine in people with T1DM and concluded that remote consultations were widely perceived as positive in people with T1DM, and most said they would consider remote consultations post-pandemic.

However, until this moment, no research has been found that has assessed adherence to online nutritional monitoring by adults with T1DM during the pandemic. However, it is expected that the participants who underwent nutritional online care during this period have maintained or adhered to better eating habits, which includes a dietary pattern rich in natural or minimally processed foods, few ultra-processed foods, and carrying out carbohydrate counting. Thus, this research aims to analyze the association between eating habits and adherence to nutritional online care by adults with T1DM during the time of social distancing because of the COVID-19 pandemic in Brazil.

## 2. Materials and Methods

### 2.1. Study Design and Participants

A cross-sectional, descriptive, and analytical study was carried out through an online form during the month of July 2020 with convenience sampling, with voluntary, anonymous participation, subject to acceptance of the informed consent form (ICF).

The study included people diagnosed with T1DM aged 18 years or older. To reinforce that the survey was aimed at people with T1DM, the survey link was sent along with a message that specified that the survey was aimed at people with T1DM and those over 18 years of age. At the beginning of the form, participants had the option to choose the condition to which they belonged; the options were: “I have type 2 diabetes”; “ I am under 18 years old and I have type 1 diabetes”; “ I am 18 or older and have type 1 diabetes”; “I have gestational diabetes”; “I have other types of diabetes”; “I am a caregiver for someone with diabetes”. If the selection did not match the expected audience (adults with T1DM), the survey was automatically closed. Therefore, those who marked some alternative other than the inclusion criteria were excluded, in addition to people who did not complete the survey or did not agree with the ICF, i.e., those who chose the option, “I do not agree with the ICF or I do not accept to participate in the research”. In that regard, 576 people responded to the questionnaire, but only 472 met the inclusion criteria.

### 2.2. Procedures

Data were collected from an online form on the Google Forms^®^ platform, in the opinion survey format, in accordance with Resolution 510/2016 [28]. The survey was disseminated and shared on the social networks, WhatsApp^®^, Instagram^®^, and Facebook^®^, and in groups aimed at people with T1DM. A pilot test was carried out with ten participants to test the time taken to complete the questionnaire and their understanding of the questions.

The following information was collected:

(a) Sociodemographic: gender; age; education (elementary school, high school, technical education, graduation, postgraduate); family income in minimum wages (MW-1045 BRL in 2020); Brazilian macro-region (North, Northeast, Midwest, Southeast, and South); situation of the city where they are living (capital, city in the metropolitan region, city in the interior of the state); and the practice of social distancing (practiced distancing, did not practice distancing);
(b) Online follow-up with health professionals in the last 30 days: (endocrinologist; general practitioner; nutritionist; nurse; psychologist; other; did not have online appointments);
(c) Eating habits during quarantine compared to the pattern before the pandemic (considering the period 30 days prior to answering the form) and carbohydrate counting:
  (c.1) Consumption of sweet foods (much higher; a little higher; the same as it was before the distancing; decreased);
  (c.2) Increased consumption of ultra-processed foods, considering frozen ready-made foods such as nuggets, pizza, or cheese bread (much higher; a little higher; equal; lower);
  (c.3) Daily consumption of fruit portions (more than five; four to five; two to three; only one; none; neither likes nor eats), considering appropriate consumption as 2 or 3 portions, and inappropriate consumption as the consumption of less than 2 portions or more than 3 portions;
  (c.4) Daily consumption of portions of vegetables and greens (more than five; four to five; two to three; only one; none; neither likes nor eats), considering appropriate consumption when consumption was equal or more than 2 portions, and inappropriate when consumption was less than 2 portions;
  (c.5) Increase in cooking habits (did not know how to cook; did not like to cook; someone else was cooking; were cooking as much as before; were cooking less than before; or were cooking more than before);
  (c.6) Number of daily meals (more than 6; between 5–6; between 3–4; between 1–2);
  (c.7) Consumption of snacks (much more than before; a little more than before; the same amount as before; less than before);
  (c.8) Delivery orders (did not order food for delivery; did order food for delivery as much as before; did order less takeout than before; did order more food for delivery than before);
  (c.9) Carbohydrate counting (does not know what it is; has heard about it but does not know how to do it; knows how to do it but does not do it; stopped doing it in social distancing; does it more often; does it at the same frequency; does it less often).

### 2.3. Data Analysis

Statistical analysis was performed using the Statistical Package for Social Science (SPSS) software. Descriptive results were expressed as absolute frequency and proportion. In the analytical stage, the variable nutritional monitoring had its categories grouped in order to classify it as “yes” or “no”, regardless of the reason. Therefore, the individuals who checked the options (a) endocrinologist; (b) general practitioner; (d) nurse; (e) psychologist; (f) other; and (g) I did not have online consultations were classified as “No”, referring to not having carried out nutritional monitoring online in the last 30 days, and individuals who checked the option (c) nutritionist were classified as “Yes”, referring to having carried out nutritional monitoring online in the last 30 days. Pearson’s chi-squared with adjusted residual analysis was applied to assess the association between carrying out nutritional care online and the factors associated with it (sociodemographic data, eating habits during quarantine compared to the pattern before the pandemic, and carbohydrate counting).

Prior to the logistic regression analysis, the absence of collinearity between the study variables was observed through linear regression, observing tolerance and variance inflation factor values, all being greater than 0.1 and less than 10, respectively. Finally, a binomial logistic regression analysis was performed, composed of the dependent variable, “did nutritional monitoring or not nutritional monitoring”, and the independent variables, consumption (or not) of fruits and did carbohydrate counting (or not) during social distancing, to determine the likelihood of adherence to online nutritional monitoring during the pandemic in contributing to better eating habits in individuals with DM1.

The final model was able to predict 86.4%. A statistical significance level of *p* < 0.05 was considered.

### 2.4. Ethical Aspects

This research was approved by the Research Ethics Committee of the Center for Tropical Medicine of the Federal University under opinion number 4.147.663 (approved on 10 July 2020) according to the Helsinki Declaration.

All the participants signed the informed consent form (ICF), agreeing to participate in the research. The identity of the participants was kept confidential, and all agreed to participate voluntarily in the research, anonymously and without financial compensation.

## 3. Results

The study included 472 individuals with a mean age of 30.24 ± 9.74 years: 86.0% were female, 47.0% lived in the Southeast macro-region of Brazil, 39.6% lived in state’s capital, 26.9% had completed higher education, and 85.6% practiced total or partial distancing.

As for the multiprofessional follow-up, 64% said they did not have online care appointments during social distancing, although among those who were assisted online, the most sought-after professionals were endocrinologists (18%), psychologists (13.1%), and nutritionists (8.9%).

No statistically significant associations were found between carrying out online nutritional monitoring and the consumption of sweet foods (*p* = 0.809), the consumption of portions of vegetables and greens (*p* = 0.081), cooking habits (*p* = 0.173), the number of daily meals (*p* = 0.758), the consumption of snacks (*p* = 0.066), and delivery orders (*p* = 0.394). No significant association was found between adherence to online nutritional monitoring and adherence to social isolation (*p* = 0.160).

In Table 1, only the data that showed a statistically significant positive or negative association are included. It was observed that living in the state capital was associated with not having received online nutritional care (*p* = 0.023), and having a family income between 10 and 20 minimum wages and living in the metropolitan region were associated with having had an online nutritional care follow-up (*p* = 0.042). As for eating habits, there was an association between having undergone an online nutritional care follow-up and a reduced consumption of ready-to-eat frozen foods (*p* = 0.021), consuming two-to-three servings of fruit per day (*p* = 0.036), and counting carbohydrates more frequently than before (*p* < 0.000), in addition to having an inverse association with consuming only one serving of fruit per day (*p* = 0.036) (Table 1).

A binomial logistic regression analysis can be seen in Table 2, composed of the dependent variable, “did nutritional monitoring or not nutritional monitoring”, and the independent variables, “consumption of fruits” and “did carbohydrate counting during social distancing”. Adhering to online nutritional monitoring during the pandemic increased the chance of doing carbohydrate counting by 2.57 times and increased the chance of eating two or more servings of fruit by 0.423 times (Table 2).

## 4. Discussion

The present study evaluated the factors associated with online nutritional care for adults with T1DM during the time of social distancing in Brazil. Most participants were female (86.0%), which corroborates the data from the study by Costa-Júnior, Couto, and Maia [29], which states that women are more concerned about body care and the prevention of diseases, dialogues about the diagnosis of the disease, ease of care, and adherence to treatment; therefore, it is believed that they are also more engaged in scientific research, collaborating with this finding.

About 64% did not carry out online care with health professionals. For nutritionists, conducting exclusively online care appointments was only authorized after the beginning of the COVID-19 pandemic [30], not previously being allowed. Among the benefits of online care during the pandemic, the easiness of appointments outside the outpatient/hospital environment, the improvement in multidisciplinary contact, and the possibility of carrying out actions of continued food and nutrition education individually or in groups stand out [14].

In the study by Russo et al. [26], the authors cite that telemedicine is an innovative approach, which has the potential to become a practical option, and that, if used correctly, can contribute to a significant improvement in the management of diabetes.

Bertuzzi et al. [31], before the pandemic, carried out a study proposing online care appointments as an innovative way of caring for people with T1DM. The authors identified that only 4 out of the 37 participants accessed online nutritional care, demonstrating that, before the pandemic, the search for online care with nutritionists was low among this population; however, the authors did not investigate the reasons for the low adherence.

Muthukrishnan et al. [32] evaluated the results of a virtual follow-up of 46 people with T1DM during the pandemic and, after 11 months, the HbA1c levels of most patients improved, pointing out that online care is a viable option that can be adopted as an additional strategy for the follow-up of these patients.

It is possible that the pandemic has influenced greater adherence to virtual care; however, despite being pointed out as a viable alternative, the lack of quality internet access becomes an obstacle. As this is an online survey, this study only reached people who had access to the internet, which may underestimate the real access and adherence to virtual services. Rozga et al. [19] mention that the main obstacle of online nutritional assistance is difficult access to the internet. Therefore, the need for investment in internet services accessible to various layers of the population is evident, in addition to facilitating access to electronic devices that enable online monitoring.

There was an association between higher income and online nutritional monitoring during the pandemic, which may be related to greater possibilities of internet access, and the possibility of paying for private online care appointments [20]. According to the Brazilian Institute of Geography and Statistics (IBGE) [33], the difference in income between households that had an internet connection and those without access is large, although the financial factor was not given as the main justification for not using the internet.

It was also observed that living in the metropolitan region was associated with online nutritional monitoring, and living in the capital was associated with not having this online monitoring. It is suggested that residents of capital cities would find it easier to get face-to-face appointments with compliance with biosafety protocols. On the other hand, people living in metropolitan regions may have opted, to a greater extent, for online appointments because they feel insecure about moving to face-to-face ones. However, further studies are needed to confirm these hypotheses.

An association was observed between the decrease in the consumption of processed foods and the daily consumption of two to three servings of fruit with adherence to online nutritional care. Furthermore, the intake of only one serving of fruit a day was associated with a lack of nutritional follow-up, perhaps because of the greater difficulty in accessing food during the research period. No studies were found that evaluated the use of online care and changes in the eating pattern of individuals, with or without T1DM, during the pandemic; however, Malta et al. [34], when analyzing the changes in the lifestyle of 45,161 Brazilian adults during social distancing, observed an increase in both genders in the consumption of ready-to-eat frozen foods. Adherence to online nutritional care increased the chance of eating fruits by 0.423 times. In a study by Grabia et al. [35], the authors evaluated the impact of the pandemic on the eating habits of patients with diabetes and observed an increase in the consumption of fresh fruit during this period.

Thus, we suggest the hypothesis that maintaining online nutritional monitoring can contribute to a greater motivation for the adoption of healthier eating habits, helping to modify the eating pattern, with less consumption of ultra-processed foods and an increased intake of food in natura, as recommended by the Guidelines of the Brazilian Society of Diabetes and by the Food Guide for the Brazilian Population [6,23]. Regarding the ingestion of only one serving of fruit a day with the lack of nutritional care, it is suggested that these people do not feel motivated or do not understand the reasons for increasing fruit consumption, or even maintain eating taboos such as the restriction of fruit consumption by people with diabetes.

Adhering to online nutritional monitoring during the pandemic increased the chance of doing carbohydrate counting by 2.57 times. CC is a gold-standard strategy that enables the development of autonomy and flexibility in food choices for individuals with T1DM; therefore, it should be encouraged [6]. However, it is a strategy that requires the emission of a series of behaviors when having meals, such as pre and post-prandial glycemic monitoring, counting the carbohydrates ingested at meals, and calculating the ultra-fast insulin dose according to the pre-prandial glycemia and measured amount of carbohydrates, which configures it as a complex practice that must be taught by a health professional, with emphasis on the nutritionist, as well as the time and motivation to perform the CC within the context of healthy eating, which may explain the association found [36]. Furthermore, as it is a complex procedure, it requires motivational strategies for adherence; therefore, in the context of behavior analysis, it is understood that changes in treatment caused by CC should increase the probability of the behavior happening again [37]. It is important to note that no studies were found that evaluated this association, even in the period prior to the pandemic.

As well as the difficulties in adhering to healthy eating habits, the practice of physical activity during social distancing also impairs glycemic control. Among the aspects of T1DM treatment, Vasconcelos et al. [38] found that social distancing and some of its consequences have impaired the practice of physical activity, affecting the habit of practicing and the characteristics of physical activity, such as frequency, duration, and intensity. The authors suggested that structured digital programs of encouragement and guidance regarding the practice of physical activity can help the population in the scenario of social distancing [39].

The present study presents limitations as being an online survey; therefore, only people with access to devices that connect to the internet participated. Moreover, the reasons why participants did not receive online nutritional care during this period were not investigated. However, this is an unprecedented study with people with T1DM, and can help to understand and reinforce the importance of maintaining the nutritional monitoring of these individuals, even online, to ensure adherence to healthy eating and, consequently, improve glycemic control. It emphasizes the need for more studies that specifically assess the effects of online nutritional monitoring in this audience.

## 5. Conclusions

This study pointed out that having performed online nutritional care during the COVID-19 pandemic was associated with reducing the consumption of ultra-processed foods, having adequate fruit consumption, counting carbohydrates more frequently, living in the metropolitan region, and having a higher income level. Therefore, online nutritional monitoring can help people with T1DM adhere to healthier eating patterns, which are of paramount importance for adequate blood glucose monitoring and the prevention of associated clinical complications.

The importance of other considerations that evaluate adherence to telenutrition consultations is also highlighted, even after the pandemic, since it is already known that the implementation of telemedicine is an innovative service that helps health care even when it is impossible to travel to a face-to-face health service, which can be a tool that monitors the patient more closely and improves adherence to nutritional treatment.

## Figures and Tables

**Table 1:** Factors associated with online nutritional monitoring for adults with type 1 diabetes mellitus during the COVID-19 pandemic in Brazil.

	Online Nutritional Monitoring n (%)		*p*-Value *
	Yes	No	
Situation of the city where living			
Capital	10 (2.1) (-)	177 (37.5) (+)	0.023 †
City in the metropolitan region	16 (3.4) (+)	92 (19.5) (-)	
City in the interior of the state	16 (3.4)	161 (34.1)	
Family income *			
<1	2 (0.4)	17 (3.6)	0.042 †
1–2	10 (2.1)	124 (26.3)	
3–5	8 (1.7)	145 (30.7)	
5–10	14 (3.0)	89 (18.9)	
10–20	8 (1.7) (+)	38 (8.1) (-)	
>20	0 (0.0)	17 (3.6)	
Consumption of ready-to-eat frozen			
Much higher	1 (0.2)	47 (10.0)	0.021 †
A little higher	8 (1.7)	92 (19.5)	
Equal	12 (2.5)	168 (35.6)	
Lower	21 (4.4) (+)	123 (26.1) (-)	
Daily consumption of fruit portions			
Neither likes nor eats	0 (0.0)	8 (1.7)	0.036 †
None	2 (0.4)	56 (11.9)	
Just 1	10 (2.1) (-)	171 (36.2) (+)	
2–3	28 (5.9) (+)	173 (36.7) (-)	
4–5	2 (0.4)	20 (4.2)	
>5	0 (0.0)	2 (0.4)	
Counting carbohydrates			
Does not know what it is	0 (0.0)	11 (2.3)	0.000 †
Has heard about it but does not know how to do it	4 (0.8)	81 (17.2)	
Knows how to do it but does not do it	2 (0.4)	63 (13.3)	
Stopped doing it in social distancing	1 (0.2)	13 (2.8)	
Does it more often	20 (4.2) (+)	75 (15.9) (-)	
Does it at the same frequency	12 (2.5)	166 (35.2)	
Does it less often	3 (0.6)	21 (4.4)	

Residual analysis: (+) significant association; (-) negative significant association; † statistical significance. *p*-value: chi-squared; * minimum wage = 1045 BRL (2020).

**Table 2:** Binomial logistic regression between online nutritional monitoring, consumption of fruits, and carbohydrate counting by adults with type 1 diabetes mellitus during social distancing due to the COVID-19 pandemic in Brazil.

	B	S.E.	Wald	*df*	Sig.	Odds Ratio	95% C.I. for EXP (B)	
							Lower	Upper
Carbohydrate counting	0.947	0.329	8.268	1	0.004	2.578	1.352	4.917
Consumption of fruits	0.861	0.278	9.578	1	0.002	0.423	0.245	0.729
Constant	2.007	0.223	80.986	1	0.000	7.443		

Binomial logistic regression. Dependent variable: online nutritional monitoring. Independent variables: consumption of fruits foods and adherence to carbohydrate counting.

## Data Availability

To request access to the data of this study, it is necessary to contact the corresponding author. Information is not available online.
